# Control of Hybrid Exciton Lifetime in MoSe_2_/WS_2_ Moiré Heterostructures

**DOI:** 10.1002/advs.202403127

**Published:** 2024-07-05

**Authors:** Haowen Xu, Jiangcai Wang, Huan Liu, Shihong Chen, Zejun Sun, Chong Wang, Rui Han, Yong Wang, Yutang Wang, Zihao Wang, Shuchun Huang, Lingwei Ma, Dameng Liu

**Affiliations:** ^1^ Institue for Advanced Materials and Technology University of Science and Technology Beijing Beijing 100083 China; ^2^ State Key Laboratory of Tribology in Advanced Equipment Department of Mechanical Engineering Tsinghua University Beijing 100084 China; ^3^ School of Resources Environment and Materials Guangxi University Nanning 530004 China; ^4^ Laboratory of Optical Detection and Imaging School of Science Qingdao University of Technology Qingdao 266033 China; ^5^ School of Mechanical Engineering and Automation Northeastern University Shenyang 110819 China

**Keywords:** electrical control, exciton‐exciton annihilation, hybrid exciton, MoSe_2_/WS_2_ moiré heterostructures, photoluminescence

## Abstract

Hybrid excitons, characterized by their strong oscillation strength and long lifetimes, hold great potential as information carriers in semiconductors. They offer promising applications in exciton‐based devices and circuits. MoSe_2_/WS_2_ heterostructures represent an ideal platform for studying hybrid excitons, but how to regulate the exciton lifetime has not yet been explored. In this study, layer hybridization is modulated by applying electric fields parallel or antiparallel to the dipole moment, enabling us to regulate the exciton lifetime from 1.36 to 4.60 ns. Furthermore, the time‐resolved photoluminescence decay traces are measured at different excitation power. A hybrid exciton annihilation rate of 8.9 × 10^−4^ cm^2^ s^−1^ is obtained by fitting. This work reveals the effects of electric fields and excitation power on the lifetime of hybrid excitons in MoSe_2_/WS_2_ 1.5° moiré heterostructures, which play important roles in high photoluminescence quantum yield optoelectronic devices based on transition‐metal dichalcogenides heterostructures.

## Graphical abstract

This study demonstrates the ability to control hybrid excitons in MoSe₂/WS₂ heterostructures by applying an out‐of‐plane electric field, tuning their lifetimes from 1.36 to 4.60 ns. Additionally, time‐resolved photoluminescence decay curves at various initial concentrations reveal the exciton‐exciton annihilation (EEA) rate of the hybrid exciton is 8.9 × 10⁻⁴ cm^2^ s⁻¹.

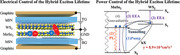

## 1 Introduction

Artificial moiré heterostructures assembled from monolayers of transition‐metal dichalcogenides (TMDC), which have distinctive electronic and optical properties, have garnered significant attention from researchers.^[^ ^1^, ^2^, ^3^ ^]^ These materials exhibit various novel physical phenomena, such as superconductivity,^[^ ^4^ ^]^ correlated insulating states,^[^ ^5^, ^6^ ^]^ and Hofstadter's butterfly physics,^[^ ^7^ ^]^ and represent a new frontier in condensed matter physics research. Among them, spatially indirect excitons are long‐lived, and can be controlled to tune their potential energy by an applied electric field or other ways,^[^ ^8^, ^9^ ^]^ which are widely used in type II TMDC heterostructures. The specific band structure of atomically thin type II heterostructures induces the spatial separation of electrons and holes into different layers.^[^ ^10^, ^11^ ^]^ This configuration renders these heterostructures an ideal platform for investigating interlayer excitons.^[^ ^12^, ^13^, ^14^ ^]^ The interlayer excitons in these structures can serve as information carriers in semiconductors. They are extensively employed in excitonic devices or circuits controlled by electric fields and power,^[^ ^15^, ^16^, ^17^ ^]^ demonstrating exceptional prospects.^[^ ^18^ ^]^

However, in contrast to intralayer excitons,^[^ ^19^ ^]^ the oscillation intensity of interlayer excitons based on type II heterostructures is weak, which limits the application of interlayer excitons. In recent years, theoretical and experimental reports have shown that there is a kind of interlayer exciton in heterostructures. The electron (hole) of this exciton is localized within one layer, while the hole (electron) is nonlocalized, forming a hybrid exciton with strong oscillation intensity and a long lifetime.^[^ ^20^, ^21^, ^22^, ^23^, ^24^ ^]^ The MoSe_2_/WS_2_ heterostructure has been proven to be an ideal platform for the study of hybrid excitons.^[^ ^25^, ^26^, ^27^, ^28^ ^]^ Due to small band spin splitting and moiré superlattices in the MoSe_2_/WS_2_ heterostructure near 0° or 60°, the intralayer and interlayer exciton can hybridize in the sample. While previous studies have shown that hybrid excitons have excellent properties, how to regulate the lifetime of hybrid excitons in this platform has not been determined, which is crucial for the further application of hybrid excitons.

In this study, we tuned the hybrid exciton lifetime in the 1.5° moiré heterostructure of MoSe_2_/WS_2_ by applying out‐of‐plane electric fields and varying the excitation power. We fabricated a dual‐gate electrical device by the dry transfer method and characterized it with Raman spectroscopy and atomic force microscopy. Then, steady‐state and time‐resolved photoluminescence (TRPL) were used to reveal the impact of the application of electric fields and excitation power on hybrid exciton lifetime in the MoSe_2_/WS_2_ heterostructure. Our results provide insights into the study of hybrid exciton lifetimes in TMDC moiré heterostructures. The study of electrically and power‐tunable hybrid exciton lifetimes is a step toward realizing efficient excitonic devices based on van der Waals heterostructures of 2D materials.

## 2 Results

### 2.1 Sample Characterization

**Figure 1:**
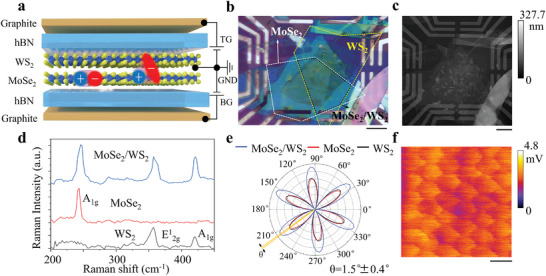
Sample characterization. a) A dual‐gated hBN‐encapsulated bilayer heterostructure MoSe_2_/WS_2_ device with graphical representations of intralayer (left) and hybrid (right) exciton species. TG and BG represent the top and bottom gate voltages applied, respectively, and GND represents the ground. b) Optical image of the entire device assembled on a SiO_2_/Si substrate. The yellow dotted contour represents WS_2_, the white dotted contour represents MoSe_2_, and the black dotted line indicates the heterostructure. The scale bar is 10 µm. c) Atomic force microscopy image of the entire device. The scale bar is 10 µm. d) Raman spectra of the single layers and heterostructure. e) Polarization‐resolved SHG intensity. The crystal orientation was determined by angle‐resolved SHG, with a twist angle close to 1.5°. f) Atomic force microscopy lateral force model showing the moiré superlattice pattern. The scale bar is 10 nm.

The device was composed of mechanically exfoliated monolayers of MoSe_2_ and WS_2_ that were completely encapsulated in hexagonal boron nitride (hBN). The top and bottom gates, as shown in **Figure** **1**a, were composed of hBN (almost the same thickness, Figure S1, Supporting Information) and a few layers of graphite in contact with prefabricated Cr/Pt metal electrodes. The entire device was assembled on a 270 nm SiO_2_ substrate by the dry transfer method. Figure 1b,c shows an optical image and an atomic force microscopy image of the entire device, respectively. The crystal orientations of MoSe_2_ and WS_2_ were determined by optical second‐harmonic generation (SHG). Figure 1e shows the SHG intensity as a function of the polarization angle of excited light in single‐layer MoSe_2_ and WS_2_, where the rotation angle approaches 1.5° and the angle misalignment is usually less than 0.5°. The red, black, and blue lines are labeled as coming from the MoSe_2_, WS_2_ monolayer, and heterostructure regions, respectively. With the twist angle approaching 0° resulting in an enhanced SHG signal.^[^ ^29^, ^30^ ^]^ Atomic force microscopy in lateral force mode is used to test the moiré superlattice periodicity of the heterostructure. Figure 1f shows the heterostructure moiré superlattice pattern, which exhibits a period of ≈6.9 nm (Figure S2, Supporting Information). Considering the influence of factors such as strain, the experimentally measured period is very close to the theoretically calculated moiré period for a 1.5° rotation angle.

To demonstrate the strong coupling of the heterostructure, Raman microscopy was used for analysis. As shown in Figure 1d, the Raman spectrum of the heterostructure region contains all peaks characteristic of the single‐layer TMDC, including the out‐of‐plane A_1g_ mode of single‐layer MoSe_2_ and the out‐of‐plane A_1g_ mode and the in‐plane E^1^_2g_ mode of single‐layer WS_2_. In addition, the intensity of the WS_2_ A_1g_ mode in the heterostructure is notably enhanced. This intensity is known to be highly sensitive to the thickness of the layer and is closely related to the degree of interlayer coupling.^[^ ^31^, ^32^ ^]^ Thus, this result indicates that the prepared heterostructure has strong interlayer coupling.

### 2.2 Emergence of Hybrid Excitons

**Figure 2:**
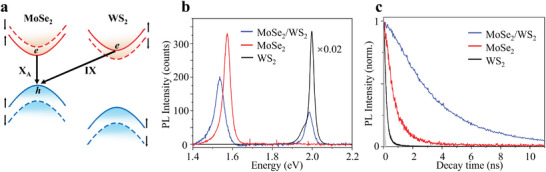
Emergence of hybrid excitons in the MoSe_2_/WS_2_ heterostructure. a) Schematic of the band structure of MoSe_2_ and WS_2_. The valence bands (blue) are staggered, and the conduction bands (red) edges are nearly degenerate. The arrows indicate the resonant hybridization between two layers with X_A_ and IX components. b) Room‐temperature photoluminescence spectra measured for single‐layer MoSe_2_ (red), WS_2_ (black), and the heterostructure (blue). c) Normalized time‐resolved photoluminescence decay curve at room temperature. The lifetime of the hybrid exciton is 3.20 ns. The gray area represents the instrument response function curve corresponding to the TRPL curve.

**Figure** **2**a shows a schematic of the direct energy gaps of a single TMDC layer semiconductor at the K and K' valleys in the Brillouin region.^[^ ^19^ ^]^ At these valleys, the lower conduction band of MoSe_2_ is closely aligned with the upper conduction band of WS_2_. Between WS_2_ and MoSe_2_, the valence band offset is large, but the conduction band offset is small,^[^ ^26^ ^]^ and the tightly aligned conduction band enhances interlayer tunneling. Therefore, the holes in the K valley can be considered to be completely localized in the MoSe_2_ layer and the electrons are not completely localized. It is the result of conduction band hybridization between the WS_2_ and MoSe_2_ layers with the same spin configuration. The hybrid excitons that form comprise both interlayer (IX) and intralayer (XA) excitons components. The hybridization caused by small angles is also accompanied by the emergence of moiré superlattices. This provides new Bragg reflections, which cause the hybrid exciton band to exhibit moiré miniband, greatly enhancing the hybridization effect.^[^ ^25^ ^]^ This enables our device to achieve hybrid excitons with both strong oscillation strength and a long lifetime.

To demonstrate the strong oscillation strength of the hybrid excitons in the device, we conducted photoluminescence (PL) tests at low excitation flow (Pth = 1 µW cm^−2^) and room temperature. As shown in Figure 2b, the PL peak in the heterostructure region includes both the intralayer exciton (XA) of WS_2_ (≈2.00 eV) and MoSe_2_ (≈1.57 eV). The stacking of WS_2_ is expected to result in a redshift of the MoSe_2_ XA exciton. Excluding hybridization, this redshift primarily originates from alterations in the dielectric environment, resulting in a small energy change of ≈10 meV.^[^ ^27^ ^]^ The hybrid exciton (≈1.53 eV) in the heterostructure exhibits a larger redshift, ≈40 meV, due to the hybridization effect at small angles. The small conduction band detuning energy in the energy band structure leads to electron tunneling at small angles, causing interlayer hybridization and the transformation of intralayer and interlayer excitons into hybrid excitons.^[^ ^21^ ^]^ When interlayer excitons hybridize with intralayer excitons through electron tunneling, the intralayer exciton components give the hybridized excitons oscillation strength.^[^ ^25^ ^]^ We observed that hybrid excitons oscillation strength is only 0.6 times weaker than that of the intralayer excitons in MoSe_2_, while the oscillation strength of interlayer excitons is generally two to three orders of magnitude weaker.^[^ ^33^ ^]^

To monitor the hybrid exciton lifetime in the MoSe_2_/WS_2_ heterostructure at room temperature, TRPL traces were obtained under low excitation intensity. Figure 2c shows the TRPL traces of the monolayers and heterostructure under low excitation flow at Pth = 1 µW cm^−2^. At low exciton concentrations, exciton‐exciton annihilation (EEA) does not occur. A 561 nm long wave pass filter was used to collect signals in the single‐layer WS_2_ region, and a bandpass filter with a central wavelength of 785 nm and a bandwidth of 60 nm was used for the single‐layer MoSe_2_ and heterostructure. For intralayer excitons and hybrid excitons, the decay curve can be well‐fitted with a single exponential function:

(1) $$I_{\left(t\right)}=A_{1}\;e^{-t/\tau_{1}}$$

where A1 and τ1 are the amplitude and decay time. According to the fitted functions, the hybrid exciton lifetime at room temperature is 3.20 ns, which is significantly longer than those of XA in the MoSe_2_ monolayer and WS_2_ monolayer (0.74 and 0.18 ns, respectively). Due to the spatial separation of electrons and holes in the heterostructure, the wavefunction overlap of the holes and electrons of the hybrid exciton decreases. Compared to the XA excitons, the hybrid excitons with IX components usually exhibits a longer lifetime, which further confirms the emergence of hybrid excitons.

### 2.3 Electrical Control of the Hybrid Exciton Lifetime

The degree of hybridization of excitons can be regulated by applying a vertical electric field, which has been demonstrated by experimental and theoretical studies.^[^ ^22^, ^28^, ^34^ ^]^ The spatial separation of electrons and holes in heterostructures gives rise to fixed out‐of‐plane dipole moments.^[^ ^35^ ^]^ This allows the spatially indirect exciton lifetime and PL emission in heterostructures can be modulated by increasing the vertical electric fields.^[^ ^36^, ^37^ ^]^ The electric fields applied (*E_appl_*) to the heterostructure are determined by a parallel plate capacitor model; the details of this approach have been reported in previous studies.^[^ ^38^ ^]^ With two nearly symmetric gates as employed in this study, the electric fields are independently introduced without doping by applying equal gate voltages with opposite signs (See Experimental Section).

**Figure 3:**
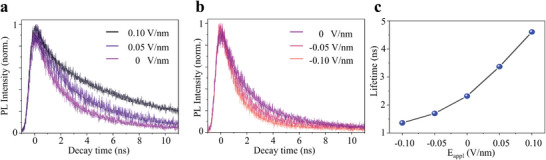
Electric field application controls the lifetime of hybrid excitons. a) TRPL decay trace under positive electric fields. Hybrid excitons exhibit more out‐of‐plane properties, with the lifetime increasing to 4.60 ns. b) TRPL decay trace under negative electric fields. The hybrid exciton exhibits more X_A_ component, with the lifetime reduced to 1.36 ns. c) Hybrid exciton lifetime versus electric fields applied.

The TRPL decay curve of the hybrid exciton under positive electric fields is shown in **Figure** **3**a. All decay curves were fitted with a single exponential function, and to avoid EEA, we conducted tests at low excitation power (P_th_ = 1 µW cm^−2^) to ensure single exponential PL decay. We observe that the lifetime of the hybrid exciton notably increases under a positive electric field, from 2.31 ns under zero electric field to 3.37 ns under 0.05 V nm^−1^ and 4.60 ns under 0.1 V nm^−1^. The observed dependence of the hybrid exciton lifetime on the electric field can be explained by the overlap changes in the tail of the electron and hole wave functions.^[^ ^36^ ^]^ Under a positive electric field, the wavefunction stretches, changing the overlap of the wavefunction tail. Therefore under an electric field antiparallel to the fixed dipole moment, electrons and holes are driven apart by the electric field, and the overlap of the wavefunction tail decreases. The hybrid exciton shows more out‐of‐plane properties, which reduces the recombination rate of the hybrid exciton and increases its lifetime.

As shown in Figure 3c, under an electric field of −0.05 V nm^−1^, the hybrid exciton lifetime decreases to 1.70 ns, lower than the lifetime (2.31 ns) under zero electric field. With increasing negative electric fields to −0.10 V nm^−1^, the hybrid exciton lifetime decreases to 1.36 ns. Therefore, when the out‐of‐plane dipole moment of the hybrid exciton is parallel to the direction of the electric field, the electrons, and holes experience an electric field force, leading to an increased overlap of their wavefunction tails. This results in more X_A_ components, accelerates the recombination rate, and leads to a reduction in the lifetime of the hybrid exciton. We investigated the variation in the hybrid exciton lifetime under positive and negative electric fields using TRPL at 77.5 K (Figure 3e). These features indicate that the hybrid exciton species in MoSe_2_/WS_2_ heterostructures are promising for further studies of electrically tunable hybrid excitons, as the development of stable and controllable approaches is imperative for realizing high‐performance exciton‐based devices.

### 2.4 Power Control of the Hybrid Exciton Lifetime

**Figure 4:**
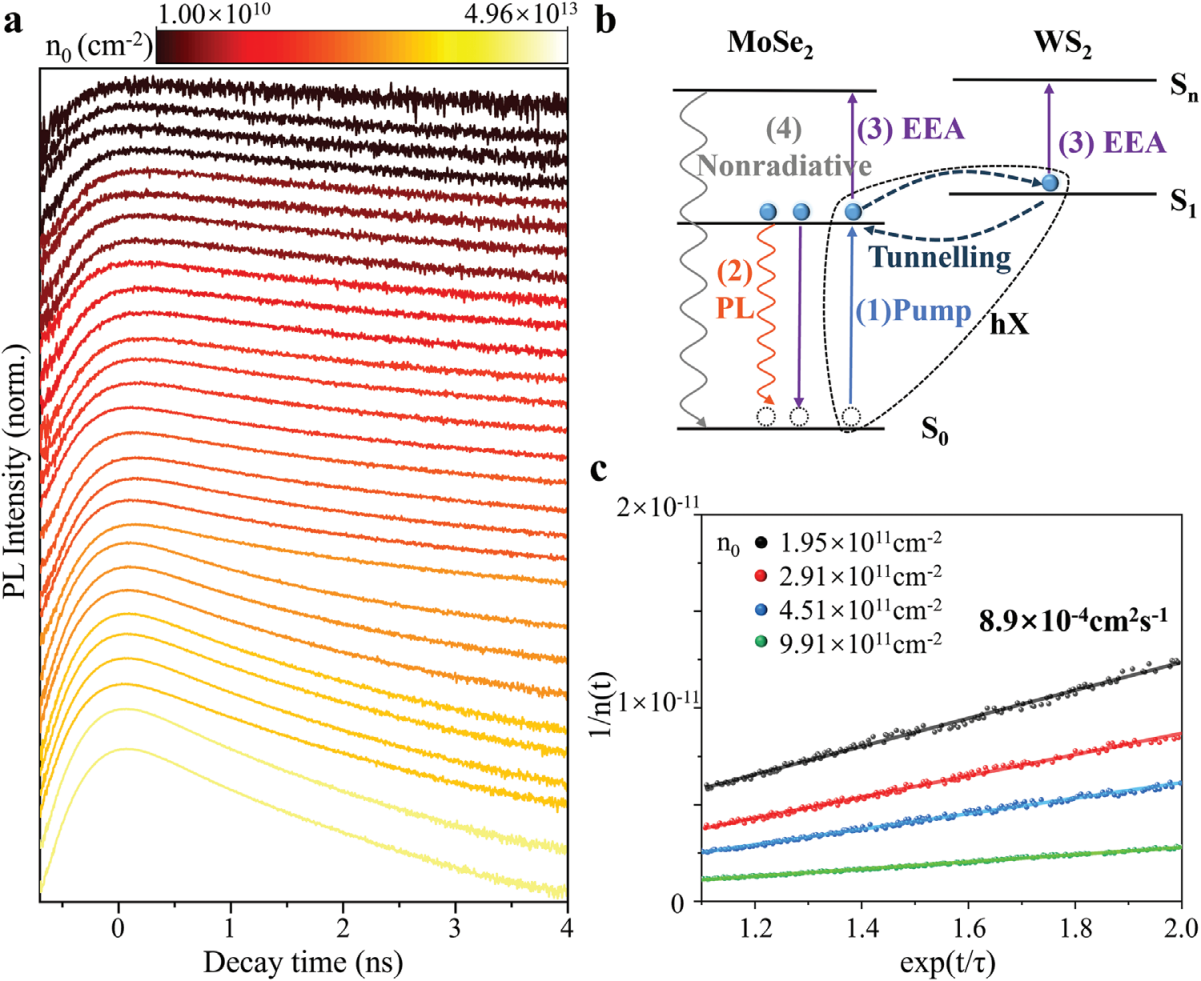
Power‐controlled hybrid exciton lifetime. a) Normalized time‐resolved PL decay curves for different initial exciton densities. The Y‐axis uses a logarithmic scale. b) Possible exciton dynamics and EEA processes. S_0_ represents the ground state; S_1_ represents the excited state; S_n_ represents high energy state. (1) The electrons are excited to the excited state S_1_; (2) Direct exciton recombination; (3) Exciton–exciton annihilation; (4) Nonradiative recombination pathways. The black dashed lines represent hybrid exciton (hX). c) Linearized data of TRPL curves using Equation (2) for the hybrid excitons in panel (a). The solid lines are linear fits.

With increasing excitation power, multi‐body interactions occur in MoSe_2_/WS_2_ heterostructures. When the energy of the higher‐order excited state is significantly greater than that of the first excited state, the occurrence of exciton‐exciton annihilation (EEA) becomes evident.^[^ ^39^ ^]^ EEA is an important factor affecting energy dissipation, where excitation energy is transferred from one exciton to another.^[^ ^40^ ^]^ This typical nonradiative compounding process is a key issue affecting the maximum efficiency of optoelectronic devices.^[^ ^41^ ^]^ TRPL kinetics is an effective method for exploring hybrid exciton annihilation. We used the pulsed laser with a wavelength of 405 nm and a repetition frequency of 40 MHz to excite the sample at a temperature of 77.5 K. A bandpass filter with a central wavelength of 785 nm and a bandwidth of 60 nm to monitor the dynamics of the hybrid excitons in the heterostructures. As shown in **Figure** **4**a, we normalized the TRPL decay curves at different initial exciton densities *n*_0_(Note S1, Supporting Information). The TRPL dynamics strongly depend on *n*_0_ and are mainly determined by the radiative recombination of hybrid excitons when *n*_0_ is low. A depopulation physical model can be utilized to depict the single‐particle and EEA processes.^[^ ^42^ ^]^ As *n*_0_ increases, the dominant exciton compound channel changes to EEA. Considering the EEA, the TRPL decay rate equation can be written as follows:^[^ ^43^ ^]^

(2) $$\frac{1}{n_{\left(t\right)}}=\left[\frac{1}{n_{0}}+\gamma /k_{0}\right]\exp\left(k_{0}t\right)-\gamma /k_{0}$$

where *n*_(*t*)_ is the number of exciton distributions, *n*_0_ is the initial exciton density, and *t* is the decay time. *k*_0_ = 1/*τ*_0_ is the intrinsic exciton recombination rate, and *τ*_0_ is the hybrid exciton lifetime at a low excitation intensity without EEA. *γ* is the exciton annihilation rate constant and is assumed to be independent of the decay time. The terms on the right of Equation (2) can describe single exciton radiative recombination and exciton‐exciton annihilation, respectively. The linear relationship of the hybrid exciton is shown in Figure 4c. To avoid complications caused by the initial peak and the instrument response function, the early portion of the TRPL, up to 0.15 ns, is omitted.^[^ ^44^ ^]^ We obtained the EEA rate *γ* of the hybrid exciton by globally fitting Equation (2) to the whole dataset, obtaining 8.9 × 10^−4^ cm^2^ s^−1^.

In Figure 4b, we summarize the possible exciton dynamics and EEA processes of hybrid excitons in heterostructure. At low excitation intensities, the electron is excited to an excited state (Equation 1). The main exciton relaxation channel is radiative recombination (Equation 2), and EEA can be neglected.^[^ ^45^ ^]^ For higher excitation intensities, the main decay channel changes to EEA (Equation 3), and energy is transferred from one exciton to another. An exciton that gains energy is excited to a high‐energy state and relaxes to a lower‐energy state through electron‐phonon interactions.^[^ ^46^, ^47^ ^]^ The excited state undergoes a nonradiative relaxation process to return to the ground state (Equation 4), resulting in the lifetime of hybrid excitons rapidly decreasing with the increase of exciton concentration (excitation power).

For monolayer TMDC, experimental measurements have shown EEA rates on the order of 10^−3^–10^−1^ cm^2^ s^−1^.^[^ ^42^, ^48^, ^49^, ^50^ ^]^ For homo or heterostructures, EEA rates on the order of 10^−5^–10^−3^ cm^2^ s^−1^ have been obtained (Table S1, Supporting Information).^[^ ^51^, ^52^, ^53^, ^54^ ^]^ The EEA rates of our 1.5° MoSe_2_/WS_2_ devices are lower than that of the TMDC monolayer but higher than that of the interlayer exciton in the moiré heterostructures. Previous works have shown that exciton lifetime is inversely proportional to the EEA rate in different excitonic materials.^[^ ^52^, ^55^ ^]^ Since the lifetime of the hybrid exciton is longer than that of X_A_ but shorter than that of IX, the EEA rate of the hybrid exciton shows a similar trend and falls between the two.

The reasons why hybrid excitons have small EEA rates can be summarized as follows. First, the hybrid excitons have the component of interlayer excitons, and the coulomb interaction makes them more ordered than the intralayer excitons.^[^ ^56^ ^]^ In this ordered arrangement, the probability of collisions between hybrid excitons will be reduced compared with intralayer excitons, resulting in a smaller EEA constant. Second, at a twist angle of 1.5°, the recombination of hybrid excitons requires the conservation of momentum and energy, while momentum requires the assistance of phonons. Therefore, the additional need for phonon assistance reduces the probability that hybrid excitons will collide and annihilate at high excitation concentrations. This results in the EEA rate of the heterostructures with angle mismatch being lower than that of the single layer.^[^ ^42^, ^57^ ^]^ Furthermore, due to the presence of moiré structures, the moiré potential binds some excitons and forms local excitons, which inhibits the EEA of hybrid excitons.^[^ ^58^ ^]^ Therefore, a small EEA rate is measured for the hybrid excitons. This provides a guide for the design of high‐quantum yield semiconductor integrated devices and light‐emitting devices based on single‐layer TMDC.

## 3 Discussion

In summary, we achieved control over the layer hybridization of hybrid excitons in MoSe_2_/WS_2_ heterostructures, allowing us to tune the hybrid exciton lifetime from 1.36 to 4.60 ns. We applied electric fields parallel (antiparallel) to the hybrid exciton dipole moment, which led to an increased (decreased) overlap of electrons and hole wavefunction tails, resulting in a faster (slower) PL decay rate. In addition, we measured TRPL decay curves at different initial concentrations and obtained a hybrid exciton EEA rate of 8.9 × 10^−4^ cm^2^ s^−1^ by fitting. This occurs because the hybrid excitons are more ordered than X_A_, the EEA process requires phonon assistance, and they are bound by the moiré potential.

Considering future circuits and devices based on exciton transport in van der Waals heterostructures, the primary factors influencing their development and application include material absorption, exciton mobility, and emission quantum yield. First, our hybrid excitons in the heterostructures, characterized by both strong oscillation intensity and long lifetime, serve as ideal carriers for information transmission in semiconductors. Second, we demonstrate the control of hybrid exciton lifetime by applying a vertical electric field through a dual‐gate structure, tuning the wave function overlap. This tunability renders hybrid excitons highly attractive for research in future electrically driven optoelectronic devices. Third, by adjusting excitation power, we achieved a small exciton‐exciton annihilation rate in the 1.5°MoSe_2_/WS_2_ heterostructure, proving the influence of power on its lifetime. This is a crucial aspect affecting the efficient modulation of light in optoelectronic devices. In addition, there are many other methods to regulate the lifetime and the degree of hybridization of hybrid excitons, such as changing the twist angle of the heterostructures,^[^ ^59^ ^]^ by applying pressure.^[^ ^60^ ^]^ It is very attractive to combine these methods to achieve further regulation of hybrid excitons in MoSe_2_/WS_2_ moiré heterostructures. These findings offer valuable insights for manipulating hybrid exciton lifetimes in MoSe_2_/WS_2_ heterostructures and designing light‐emitting devices based on 2D materials.

## 4 Experimental Section

### Sample Preparation and Device Fabrication

Monolayer MoSe_2_ and WS_2_ flakes were obtained by mechanical exfoliation from bulk crystals (HQ graphene). The atomically thin flakes were exfoliated from bulk crystals onto oxidized Si substrates. The identification of monolayer WS_2_ and MoSe_2_ flakes was based on their optical contrast. The crystal orientations of WS_2_ and MoSe_2_ were determined through optical second‐harmonic generation (SHG). The thin layers of graphite and hBN were also mechanically exfoliated onto the silicon wafer, and their thicknesses were confirmed using AFM. A transparent stamp made of polydimethylsiloxane (PDMS) coated with polypropylene carbonate (PC) was used to pick up the few layers of hBN. Then, hBN was used to pick up the TMDC layers using a transfer microscope with precise rotation control. All the devices were released from the stamp onto SiO_2_ substrates with pre‐patterned Cr/Pt metal electrodes at ≈120 °C. The polycarbonate residue on the devices was removed by dissolving it in chloroform.

### Optical Measurement

The region of the MoSe_2_/WS_2_ heterostructures was identified using Micro‐Raman spectroscopy and photoluminescence spectroscopy (Evolution, Horiba) with a 532 nm wavelength laser (photon energy of 2.33 eV). For time‐resolved PL measurements in electrical control and power control sections, the sample was placed in a continuous‐flow liquid helium cryostat (RC102‐CFM, CIA) and cooled to 77.5 K. The sample was excited with a pulsed laser at 405 nm wavelength (photon energy of 3.06 eV) and 40 MHz repetition frequency. The laser was focused on the samples through a 100X objective lens (LMPLFLN 100 X, 0.8 NA, Olympus). The same objective was used to collect PL signals. The PL signals went through a long‐pass filter or a band filter (Semrock) and were detected by a single‐photon avalanche photodiode (SPD500‐S, SIMTRUM). Time‐resolved photoluminescence traces were acquired using a time‐correlated single‐photon counting system (Multiharp 150, Pico Quant). The data were analyzed with software (SymPhoTime 64). All cryogenic measurements were performed at 77.5 K under vacuum.

### Electrostatic Model for Hybrid Excitons in TMDC Bilayers

The applied electric fields *E*_appl_ to the entire device were calculated by:

(3) $$E_{appl}=\frac{V_{tg}-V_{bg}}{t_{total}}\;$$

The local electric field *E*_hs_ in the double layer at an applied electric field was calculated based on the parallel plate capacitor model:

(4) $$E_{hs}=E_{appl}\;\cdot \frac{\varepsilon_{hBN}}{\varepsilon_{TMDC}}$$

where *V_tg_* and *V_bg_* represent the top and bottom gate voltages, respectively; *t_total_* is the thickness of the hBN dielectric layer; and ε_*hBN*_ ≈3.5 and ε_*TMDC*_ ≈7 are the dielectric constants of the hBN and TMDC layers, respectively.^[^ ^61^ ^]^ Two almost symmetric dual‐gate structures were used to independently apply vertical electric fields to the hetero‐bilayer. With the application of equal gate voltages with opposite signs (*V_tg_* = −α*V_bg_*), the electric fields were independently introduced without doping, where α ≈ 1 is the ratio of the thicknesses of the top and bottom hBN layers. The gate voltages were applied by source meters (Model 2400, Keithley).

### Angle‐Resolved Optical SHG

The crystal orientations of the WS_2_ and MoSe_2_ monolayers were determined through second harmonic generation (SHG) measurements using an optical microscope (IX3‐LHLEDC, Olympus). A linearly polarized femtosecond laser light (800 nm, 80 fs, 3.5 W, Coherent Chameleon) was focused onto the monolayers with an objective lens (LMPLFLN 100 X, 0.8 NA, Olympus). The reflected SHG signals were collected by the same objective lens, which passes through a short‐pass dichroic mirror, Glan–Taylor linear polarizer. The signals were focused by a 4X objective (0.13 NA, Olympus) onto a fiber (M28L02, Thorlabs) and through a fiber coupler to be separated by a spectrometer (HORIBA, IHR550) and detected by an EMCCD camera (Synapse EM, Horiba).

### AFM

An atomic force microscope from Asylum Research (Cypher S, Asylum Research) was used to perform the moiré superlattice measurements with a silicon AFM tip (ContDLC, BudgetSensors). For the entire device, topographic imaging was performed using an atomic force microscope (Dimension Icon, Bruker) with the tip (Asyelec‐01‐R2, Asylum Research). All the measurements were made under stable environmental conditions (≈20 – 25 °C, relative humidity 20 to 30%).

## Conflict of Interest

The authors declare no conflict of interest.

## Author Contributions

H.X. and J.W. contributed equally to this work. H.X. and J.W. performed conceptualization, data curation, wrote the original draft, and reviewed and edited the final manuscript. H.L. performed conceptualization and project administration. S.C., C.W., and Y.W. acquired resources. Z.S performed validation. R.H. performed visualization. Y.W. performed funding acquisition. Z.W. performed methodology. S.H. wrote the original draft. L.M. performed Supervision. D.L. performed funding acquisition and supervision.

## Supporting information

Supporting Information

## Data Availability

The data that support the findings of this study are available from the corresponding author upon reasonable request.
