# Supplementary material for: Control of Hybrid Exciton Lifetime in MoSe2/WS2 Moiré Heterostructures
Source: Adv Sci (Weinh). 2024 Jul 5;11(34):2403127. doi: 10.1002/advs.202403127 (PMC11425870; doi:10.1002/advs.202403127)
Supplement: Supplementary file 1 — Supporting Information [file ADVS-11-2403127-s001.docx]

Supporting Information For

**Control of hybrid exciton lifetime in MoSe_2_/WS_2_ moiré heterostructures**

Haowen Xu^1, #^, Jiangcai Wang^2, #^, Huan Liu^2, *^, Shihong Chen^3^, Zejun Sun^2^, Chong Wang^2^, Rui Han^2^, Yong Wang^5^, Yutang Wang^4^, Zihao Wang^3^, Shuchun Huang^2^, Lingwei Ma^1, *^, Dameng Liu^2, *^

^1^Institue for Advanced Materials and Technology, University of Science and Technology Beijing, Beijing 100083, China.

^2^State Key Laboratory of Tribology in Advanced Equipment, Department of Mechanical Engineering, Tsinghua University, Beijing 100084, China.

^3^School of Resources, Environment and Materials, Guangxi University, Nanning 530004, China.

^4^School of Mechanical Engineering and Automation, Northeastern University, Shenyang 110819, China.

^5^Laboratory of Optical Detection and Imaging, School of Science, Qingdao University of Technology, Qingdao 266033, China.

^#^These authors contributed equally to this work.

**Corresponding Author**

*E-mail:

liuhuan122@mail.tsinghua.edu.cn; mlw1215@ustb.edu.cn; ldm@tsinghua.edu.cn


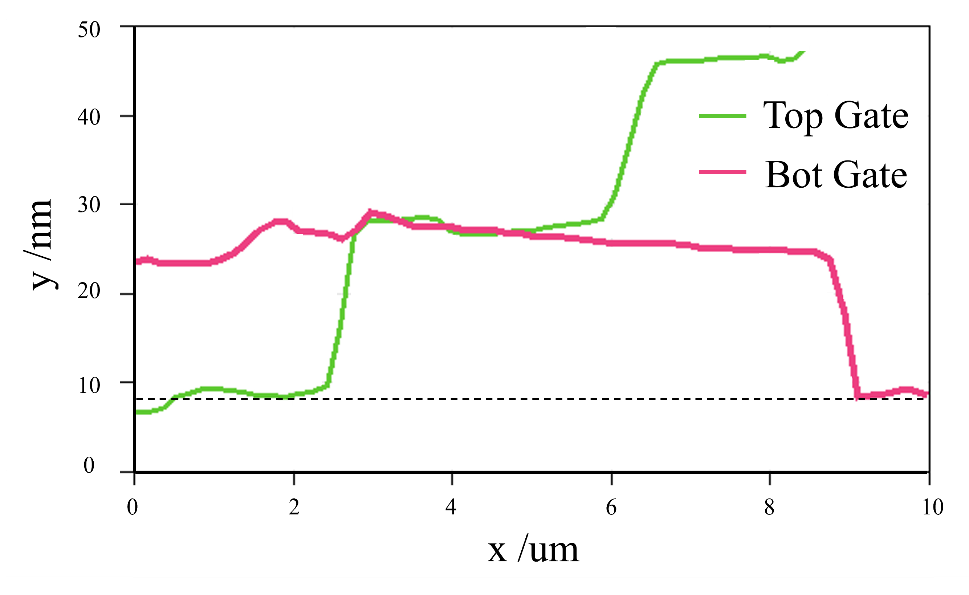


**Figure S1.** The thickness of the hBN dielectric layer is 19nm at both the top and bottom, related to **Figure 1c**.


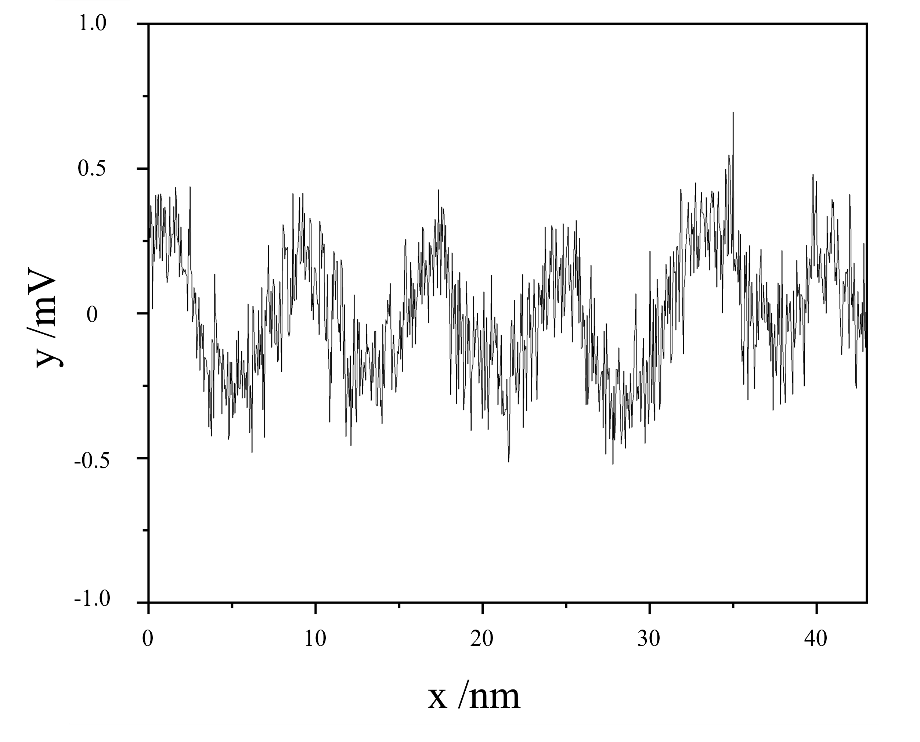


**Figure S2.** The period of the moiré lattice. It is about 6.9nm, related to **Figure 1e**. The period of the moiré lattice can be approximated as: $\text{a}_{\text{m}}\text{=}\text{a}_{\text{se}}\text{/}\sqrt{\text{δ}^{\text{2}}\text{+}\text{ξ}^{\text{2}}}$, where$\text{ }\text{a}_{\text{se}}\text{=0.328}$ and $\text{a}_{\text{s}}\text{=0.315}$ are the lattice constants of MoSe_2_ and WS_2_ respectively;^[1]^ $\text{ξ}$ is the torsion angle of the two layers; $\text{δ}\text{≈4\%}$ is the lattice mismatch constant.


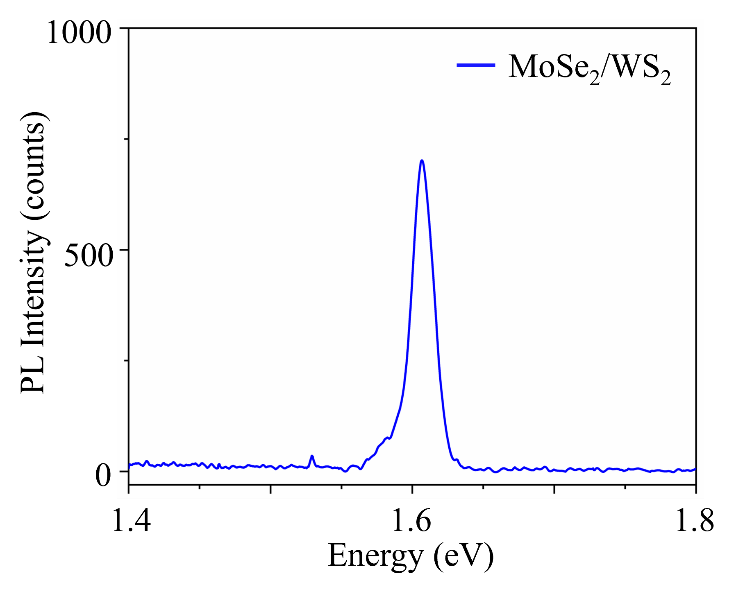


**Figure S3.** PL spectra of MoSe_2_/WS_2_ heterostructure. The heterostructure has a temperature of 77.5 K and is excited by a steady-state laser with a wavelength of 405 nm and an excitation power of 1.2 µW. The photoluminescence peak at 1.61 eV is labeled from hybrid excitons.

**Table S1.** Exciton-exciton annihilation (EEA) rate table

|  | EEA rate | Temperature | Reference |
| --- | --- | --- | --- |
| Monolayer WS_2_ | 0.41 ± 0.02 | Room Temperature | Ref. ^[2]^ |
| Bilayer WS_2_ | 6 ± 1.09×10^-3^ | Room Temperature |  |
| Trilayered WS_2_ | 1.88 ± 0.47×10^-3^ | Room Temperature |  |
| Monolayer WSe_2_ | 5×10^-2^ | Room Temperature | Ref.^[3]^ |
| Bilayer WSe_2_-1° | 2.6×10^-2^ | Room Temperature | Ref.^[4]^ |
| Bilayer WSe_2_-32° | 5.3×10^-2^ | Room Temperature |  |
| Bilayer WSe_2_-60° | 1.9×10^-2^ | Room Temperature |  |
| Monolayer MoSe_2_  - unencapsulated | 3.3×10^-1^ | Room Temperature | Ref.^[5]^ |
| Monolayer MoSe_2_  - encapsulated | 3×10^-3^ | Room Temperature |  |
| Monolayer MoS_2_  - encapsulated | 1×10^-3^ | Room Temperature | Ref.^[6]^ |
| MoSe_2_/WSe_2_ heterobilayers | 5×10^-3^ | 5K | Ref.^[7]^ |
| WS_2_/WSe_2_ moiré heterobilayers - 58° | 1.3×10^-5^ | 4K | Ref.^[8]^ |

**Note 1.** Estimation of carrier density under pulsed excitations:

Under pulse excitation, the carrier density injected per pulse (cm^-2^):

$$\begin{aligned} \text{n}_{\text{0}}\text{=}\frac{\text{Pσ}}{\text{AfE}}\#\left（ 1.1 \right） \end{aligned}$$

where *P* is the laser power (W); *σ* is the absorption coefficient (the absorption rate of incident power); *A* is the spot area of the pulsed laser on the focal plane of the sample (cm^-2^); *f* is the pulse repetition frequency (s^-1^); and *E* is the photon energy (J).

**Note 2.** Exciton-exciton annihilation (EEA) rate fitting

Taking into account the EEA, the rate equation of TRPL decay can be written as:

$$\begin{aligned} \frac{\text{d}\text{n}\left( \text{t} \right)}{\text{dt}}\text{=-}\text{k}_{\text{0}}\text{n}\left( \text{t} \right)\text{-}\text{γn}\left( \text{t} \right)^{\text{2}}\#(2.1) \end{aligned}$$

The solution to Eqn. (1) is given by：

$$\begin{aligned} \text{n}\left( \text{t} \right)\text{=}\frac{\text{n}_{\text{0}}\exp\left( \text{-}\text{k}_{\text{0}}\text{t} \right)}{\text{1+}\left( \text{γ}/{\text{k}_{\text{0}}} \right)\text{n}_{\text{0}}\left[ \text{1-}\exp\left( \text{-}\text{k}_{\text{0}}\text{t} \right) \right]}\#(2.2) \end{aligned}$$

Eqn. (2) can be linearized as：

$$\begin{aligned} \frac{\text{1}}{\text{n}_{\left( \text{t} \right)}}\text{=}\left[ \frac{\text{1}}{\text{n}_{\text{0}}}\text{+}\text{γ}/{\text{k}_{\text{0}}} \right]\exp\left( \text{k}_{\text{0}}\text{t} \right)\text{-}\text{γ}/{\text{k}_{\text{0}}}\#(2.3) \end{aligned}$$

The EEA rate *γ* is obtained by solving the slope of Eqn. (3), where k_0_=1/τ_0_ is the exciton recombination rate without EEA. The right side of Eqn. (3) describes the exciton radiation recombination and EEA process.

**References:**

[1] Y. Tang, L. Li, T. Li, Y. Xu, S. Liu, K. Barmak, K. Watanabe, T. Taniguchi, A. H. MacDonald, J. Shan, K. F. Mak, *Nature* **2020**, 579, 353.

[2] L. Yuan, L. Huang, *Nanoscale* **2015**, 7, 7402.

[3] M. Massicotte, F. Vialla, P. Schmidt, M. B. Lundeberg, S. Latini, S. Haastrup, M. Danovich, D. Davydovskaya, K. Watanabe, T. Taniguchi, V. I. Fal’ko, K. S. Thygesen, T. G. Pedersen, F. H. L. Koppens, *Nat. Commun.* **2018**, 9, 1633.

[4] Y. Chen, B. Cao, C. Sun, Z. Wang, H. Zhou, L. Wang, H. Zhu, *Nano Research* **2022**, 15, 4661.

[5] C. P. Walsh, J. P. Malizia, S. C. Sutton, J. M. Papanikolas, J. F. Cahoon, *Nano Lett.* **2024**, 24, 1431.

[6] A. Steinhoff, F. Jahnke, M. Florian, *Physical Review B* **2021**, 104, 155416.

[7] E. Wietek, M. Florian, J. Göser, T. Taniguchi, K. Watanabe, A. Högele, M. M. Glazov, A. Steinhoff, A. Chernikov, *Physical Review Letters* **2024**, 132, 016202.

[8] C.-S. Cai, W.-Y. Lai, P.-H. Liu, T.-C. Chou, R.-Y. Liu, C.-M. Lin, S. Gwo, W.-T. Hsu, *Nano Letters* **2024**.
